# Retinal Microvascular Impairment in COVID-19 Bilateral Pneumonia Assessed by Optical Coherence Tomography Angiography

**DOI:** 10.3390/biomedicines9030247

**Published:** 2021-03-02

**Authors:** Jorge González-Zamora, Valentina Bilbao-Malavé, Elsa Gándara, Anna Casablanca-Piñera, Claudia Boquera-Ventosa, Manuel F. Landecho, Javier Zarranz-Ventura, Alfredo García-Layana

**Affiliations:** 1 Department of Opthalmology, Clínica Universidad de Navarra, 31008 Pamplona, Spain; jgzamora@unav.es (J.G.-Z.); vbilbao@unav.es (V.B.-M.); egandararod@unav.es (E.G.); 2 Institut Clínic de Oftalmología (ICOF), Hospital Clínic de Barcelona, 08028 Barcelona, Spain; casablancanna@gmail.com (A.C.-P.); boquera.claudia@gmail.com (C.B.-V.); 3 COVID-19 Unit, Clínica Universidad de Navarra, 31008 Pamplona, Spain; mflandecho@unav.es; 4 Department of Internal Medicine, Clínica Universidad de Navarra, 31008 Pamplona, Spain; 5 Institut de Investigacions Biomediques August Pi i Sunyer (IDIBAPS), 08036 Barcelona, Spain

**Keywords:** SARS-COV-2, COVID-19, coronavirus, retina, microvascular, cotton wool spot, optical coherence tomography, OCT, optical coherence tomography angiography, OCTA

## Abstract

The purpose of this study was to evaluate the presence of retinal and microvascular alterations in COVID-19 patients with bilateral pneumonia due to SARS-COV-2 that required hospital admission and compare this with a cohort of age- and sex-matched controls. COVID-19 bilateral pneumonia patients underwent retinal imaging 14 days after hospital discharge with structural optical coherence tomography (OCT) and optical coherence tomography angiography (OCTA) measurements. Vessel density (VD) and foveal avascular zone (FAZ) area were evaluated in the superficial, deep capillary plexus (SCP, DCP), and choriocapillaris (CC). After exclusion criteria, only one eye per patient was selected, and 50 eyes (25 patients and 25 controls) were included in the analysis. COVID-19 patients presented significantly thinner ganglion cell layer (GCL) (*p* = 0.003) and thicker retinal nerve fiber layer (RNFL) compared to controls (*p* = 0.048), and this RNFL thickening was greater in COVID-19 cases with cotton wool spots (CWS), when compared with patients without CWS (*p* = 0.032). In both SCP and DCP, COVID-19 patients presented lower VD in the foveal region (*p* < 0.001) and a greater FAZ area than controls (*p* = 0.007). These findings suggest that thrombotic and inflammatory phenomena could be happening in the retina of COVID-19 patients. Further research is warranted to analyze the longitudinal evolution of these changes over time as well as their correlation with disease severity.

## 1. Introduction

The current coronavirus outbreak, also known as COVID-19 syndemic, was first reported in Wuhan, China, in December of 2019 and secondarily spread. COVID-19 is caused by SARS-COV-2, a novel betacoronavirus which causes a life-threatening infection that has caused more than 1 million deaths worldwide. To enter human cells, SARS-COV-2 uses a spike protein which directly binds with a strong affinity to human angiotensin converting enzyme 2 (ACE2) [1]. COVID-19 might induce a severe acute respiratory distress syndrome, associated with a prothrombotic state and a multiorgan failure with a high mortality rate in some cases. 

It is noteworthy that the prothrombotic state is clinically relevant even in patients who had received prior thromboprophylaxis [2]. In fact, microvascular endothelial injury, combined with cytokine overproduction, have been hypothesized as the key factor leading patients with severe COVID-19 to multiple organ failure [3]. In this sense, there has been recent histological evidence of SARS-COV-2 inside human endothelial cell. Findings from postmortem studies have shown the presence of viral elements within kidney endothelial cells and an accumulation of inflammatory cells, suggestive of endothelitis, in samples from lungs, heart, kidneys, and small intestine [4]. In addition, viral particles within cytoplasmic vacuoles of capillary endothelial cells have been also demonstrated by electron microscopy sections of brain tissue samples from a patient with SARS-COV-2 severe acute respiratory syndrome [5]. 

The vascular endothelium plays an essential role in the control of vascular tone and the preservation of the blood-retinal barrier, through its active paracrine, endocrine, and autocrine functions. Endothelium dysfunction is a major contributor to microvascular ischemia by shifting the vascular balance toward greater vasoconstriction with consequent inflammation, tissue edema, and pro-coagulant state [6,7]. In vivo noninvasive assessment of microvascular function can be analyzed on few organs, such as skin, bulbar conjunctiva, sublingual mucosa, and retina [8]. The first study that evaluated the microvascular blood flow in patients with SARS-COV-2 severe pneumonia, through the sublingual microcirculation, found an inverse correlation between perfused vessel density and D-dimer levels, which supports the association between coagulopathy and microvascular perfusion disturbances in patients with severe SARS-COV-2 [9]. 

The human eye has its own renin-angiotensin system which is not only present in the surface of the eye but also in the retina [10,11]. In the eye, the retinal macrovasculature (central retinal artery and vein major branches) has been analyzed in COVID-19 patients [12]. The analysis of the microvasculature can be done in a non-invasive way with new technologies such as optical coherence tomography angiography (OCTA), which is capable of quantifying blood flow in the different capillary plexi: the superficial capillary plexus (SCP) and the deep capillary plexus (DCP), which compose the internal blood-retinal barrier, and the choriocapillaris (CC) [13].

Coronaviruses are capable of producing a wide spectrum of ocular signs and symptoms and these commonly appear in patients with severe pneumonia [14]. However, only few studies have described retinal manifestations [12,15,16,17,18], due to the need of keeping patients in isolation and the difficulty of performing complex tests without increasing the risk of contagion. Moreover, some of the findings reported have been controversial and questioned as a possible misinterpretation of the retinal images [19,20]. To date, no consistent data is available in the literature on the microvascular complications of COVID-19. 

The aim of this study is to perform in a series of COVID-19 bilateral pneumonia patients, that required hospital admission, a comprehensive ocular examination with retinal imaging techniques including fundus retinographies, structural OCT and OCTA, to evaluate the status of the perifoveal vascular network and the integrity of the internal blood-retinal barrier, and to compare this with a subset of age- and sex-matched healthy controls from a larger cohort. This study is directed to investigate the potential of OCTA to address this possible microvascular impairment in an objective manner and to ascertain the role of OCTA to identify patterns of disease that may help in the systemic management of these patients.

## 2. Materials and Methods

### 2.1. Study Design and Ethics Approval

Cross-sectional, consecutive case-control series. Cases were selected from a series of COVID-19 bilateral pneumonia patients, with at least one SARS-COV-2 positive polymerase chain reaction (PCR) test, admitted at the Clínica Universidad de Navarra (Pamplona, Spain) during March and April 2020. All patients were invited to participate in the study 14 days after hospital discharge, and a total of 30 subjects agreed (*n* = 30; eyes = 60). At the moment of ocular examination, all patients were asymptomatic and had negative results from oral and nasopharyngeal swab and positive antibodies. Controls were obtained from a large OCTA database (*n* = 1186) at Hospital Clinic (Barcelona, Spain) from previous research projects (ClinicalTrials.gov NCT03422965) [21,22]. This project was approved by the Institutional Review Board of Clínica Universidad de Navarra and Hospital Clinic of Barcelona (study codes 2020.224 approved on 9 November 2020 and HCB/2016/0216 approved on 16 December 2016 respectively) and followed the tenets set forth in the Declaration of Helsinki. Written informed consent was obtained for all participants (cases and controls).

### 2.2. Inclusion and Exclusion Criteria

**Figure 1:**
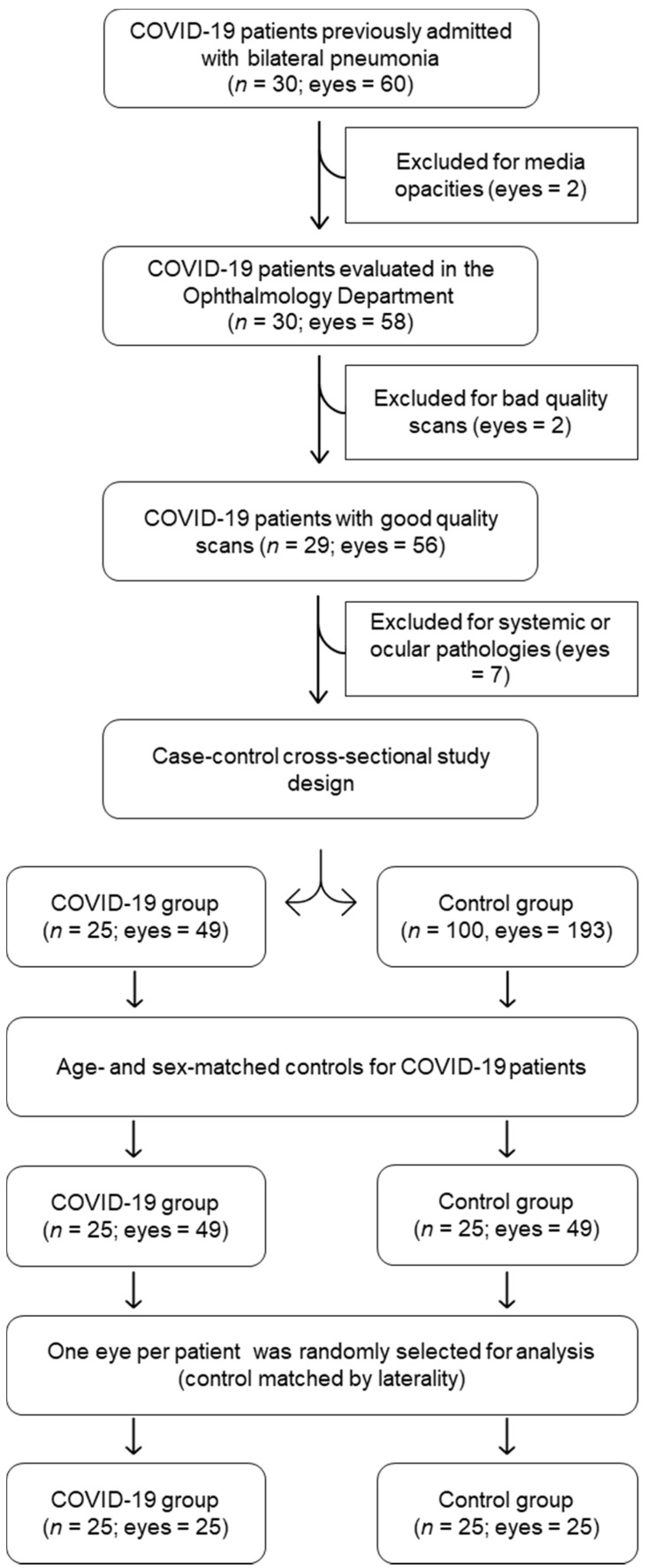
Consolidated standard for reporting trials (CONSORT)-style flow chart describing included and excluded eyes in the study.

In the case cohort, those presenting media opacities such as cataract and vitreous haemorrhages (eyes = 2), poor quality scans (eyes = 2), or concomitant systemic or ocular pathologies such as diabetes mellitus, glaucoma, high myopia, fovea plana, and age-related macular degeneration (eyes = 7) were excluded from analysis (*n* = 25; eyes = 49). In the control cohort, the same exclusion criteria were applied and a total sample of 193 eyes from 100 healthy volunteers were included in the study. To eliminate the influence of demographic factors, a subset of age- and sex-matched controls were randomly selected (1:1) for comparison of measurements. To avoid a potential risk of bias due to bilaterality of cases, only one eye of each of these patients was randomly selected and included in the case-control study (*n* = 25; eyes = 25, for each cohort). A consolidated standard for reporting trials (CONSORT)-style flow chart describing included and excluded eyes is presented in Figure 1. 

### 2.3. Ocular Examination, OCT, and OCTA Imaging Protocols

All participants underwent a complete ocular examination. Clinical data collected included best-corrected visual acuity (BCVA), slit-lamp biomicroscopy, intraocular pressure measurement, and retinal fundus examination. Qualitative findings observed in fundus examination included presence/absence of hard exudates and cotton wool spots (CWS). All fundus retinographies, OCT, and OCTA images were obtained using the same device (DRI OCT Triton SS-OCT Angio, Topcon Medical Systems, Inc. Oakland, NJ, USA) in both study centers. Structural OCT images were captured using the 3D Wide 12 × 9 mm and the 3D Disc 6 × 6 mm scanning protocols, and OCTA images were captured using the 4.5 × 4.5 mm and the 6 × 6 mm scanning protocols.

### 2.4. Structural OCT and OCTA Analysis

**Figure 2:**
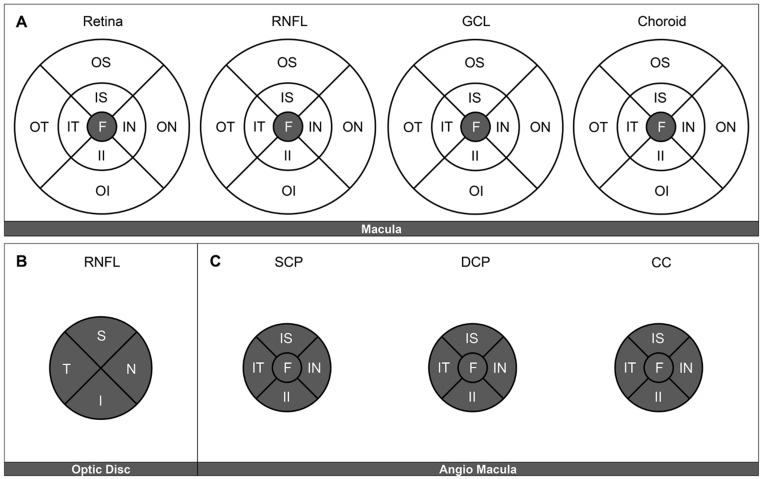
Regions of interest in the macula, defined by the early treatment diabetic retinopathy (ETDRS) grid subfields. The evaluated fields on each of the structural optical coherence tomography (OCT) and OCT angiography (OCTA) are presented in gray (**A**). Structural OCT, macula. Central ETDRS subfield measurements of foveal, retinal nerve fiber layer (RNFL), ganglion cell layer (GCL), and choroid thickness, respectively (**B**). Structural OCT, optic nerve head. Four quadrants (**C**). OCTA, central and inner ring ETDRS subfields. Vessel density measurements were obtained in the superficial capillary plexus (SCP), deep capillary plexus (DCP), and choriocapillaris (CC) layers. F: fovea. IS: inner superior. IN: inner nasal. II: inner inferior. IT: inner temporal. OS: outer superior. ON: outer nasal. OI: outer inferior. OT: outer temporal. S: superior. N: nasal. I: inferior. T: temporal.

Structural OCT macular parameters were measured using the early treatment diabetic retinopathy (ETDRS) grid centered in fovea by fixation. Three areas of interest were defined as fovea (central subfield), inner ring (inner ring subfields), and outer ring (outer ring subfields). The retinal stratus and parameters analyzed were total retina, retinal nerve fiber layer (RNFL), ganglion cell layer (GCL), and choroid thicknesses as delineated by the boundaries automatically defined by the built-in segmentation software in the commercial device (Figure 2). Optic nerve head RNFL thickness was measured in each of the four quadrants using a radial scan centered on the optic nerve head and presented as mean RNFL. 

OCTA parameters evaluated were vessel density (VD) in the three different plexi: SCP, DCP, and CC, also using the ETDRS grid subfields to define the areas of interest. Mean VD was calculated as the average value obtained in the fovea (central ETDRS subfield) and parafoveal area, defined as the area conformed by the inner superior (IS), inner nasal (IN), inner inferior (II), and inner temporal (IT) ETDRS subfields centered on the macula by fixation. The foveal avascular zone (FAZ) area was manually delineated on the SCP and the DCP by two independent graders, encompassing the central fovea where there were no clear and demarcated vessels seen on the OCTA. 

### 2.5. Statistical Analysis

Clinical demographics and imaging data were analyzed with frequency and descriptive statistics. The description of quantitative variables was performed using the mean, standard error of the mean (SEM), median, and quartiles. The Kolmogorov–Smirnov test was used to assess the normality of distributions. Clinical variables, structural OCT, and OCTA parameters were compared between cases and controls and between COVID-19 patients with and without CWS on the posterior pole using the Student’s *t*-test. Categorical variables were presented as percentages, and comparison between groups were performed with the chi-square test. For all the tests, *p* values < 0.05 were considered statistically significant. All statistical analyses were performed using GraphPad Prism version 5.0 software (GraphPad Software Inc., San Diego, CA, USA).

## 3. Results

After applying all the filters, a highly selected cohort of 50 eyes from 25 COVID-19 bilateral pneumonia cases and 25 age- and sex-related healthy controls was included in the analysis. Each group was composed of 11 right eyes and 14 left eyes and included 11 women and 14 men. The mean age of the cases and controls was 61.02 (±1.64) and 59.62 (± 1.61) years of age, respectively (*p* = 0.69). The demographics and baseline characteristics of both study cohorts are described in detail in Table 1.

### 3.1. Structural OCT Outcomes

**Figure 3:**
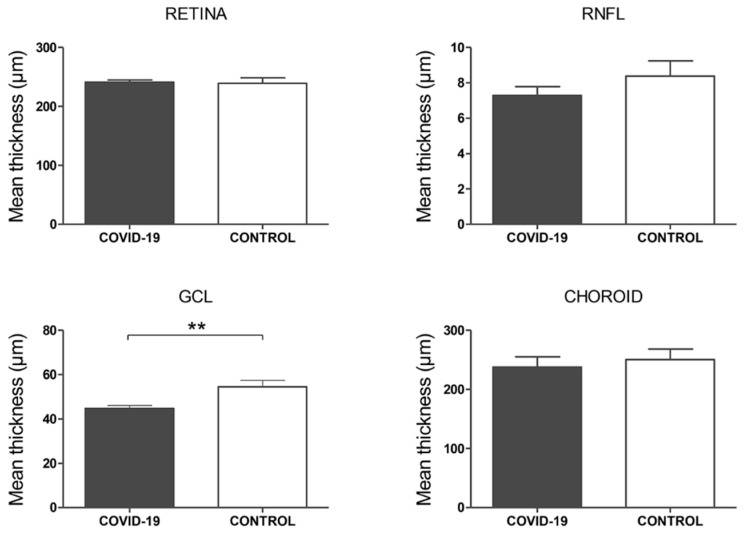
Structural optical coherence tomography (OCT) analysis, macula. Mean foveal thickness (central subfield of the ETDRS grid) in microns of the retina, retinal nerve fiber layer (RNFL), ganglion cell layer (GCL), and choroid layers. *p*-values correspond to a comparison between COVID-19 patients and controls. Error bars correspond to the SEM. ** *p* < 0.01.

In the structural OCT analysis of macular compartments, a significantly thinner GCL was observed in COVID-19 cases compared to controls (44.8 ± 1.32 vs. 54.4 ± 2.90, *p* = 0.003). However, no differences were found in central retinal (241.1 ± 3.77 vs. 239.2 ± 9.38, *p* = 0.85), RNFL (7.3 ± 0.50 vs. 8.38 ± 0.86, *p* = 0.28), and choroidal thickness (237.6 ± 17.83 vs. 250.5 ± 17.73, *p* = 0.60) (Figure 3). It is important to clarify that, when present, CWS were located outside the macular area, so the possibility of projection artifacts in the OCT was discarded.

**Figure 4:**
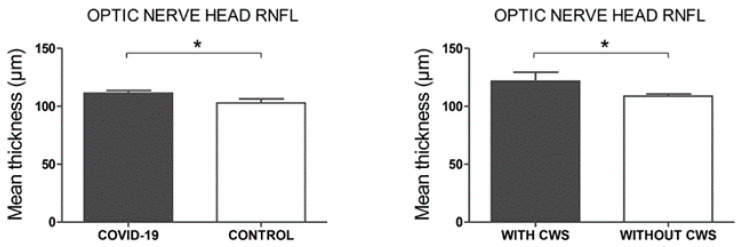
Structural optical coherence tomography (OCT) analysis, optic nerve head. Mean thickness in microns of the retinal nerve fiber layer (RNFL). Left *p*-values correspond to a comparison between COVID-19 patients and controls. Right *p*-values correspond to a comparison between COVID-19 patients with cotton wool spots (CWS) and without CWS. Error bars correspond to the SEM. * *p* < 0.05.

In the optic nerve head analysis, a significantly thicker mean RNFL was observed in Covid-19 cases compared to controls (111.4 ± 2.31 vs. 103.1 ± 3.45, *p* = 0.048) (Figure 4). All these results are described in detail in Table 2.

In the subgroup analysis by presence of CWS within the COVID-19 cases cohort, none of the macular parameters showed differences between groups. In the optic nerve head analysis, of the five patients (eyes = 6) that presented CWS on the posterior pole the day of the ophthalmologic examination (20%), one eye was excluded of this analysis because the CWS was localized in that area and prevented adequate segmentation of the RNFL. Significantly thicker RNFL was observed in the COVID-19 patients with CWS compared to those that showed no CWS (121.8 ± 7.62 vs. 109.0 ± 1.81, *p* = 0.032) (Figure 4). These results are presented in Table 3.

### 3.2. OCTA Outcomes

**Figure 5:**
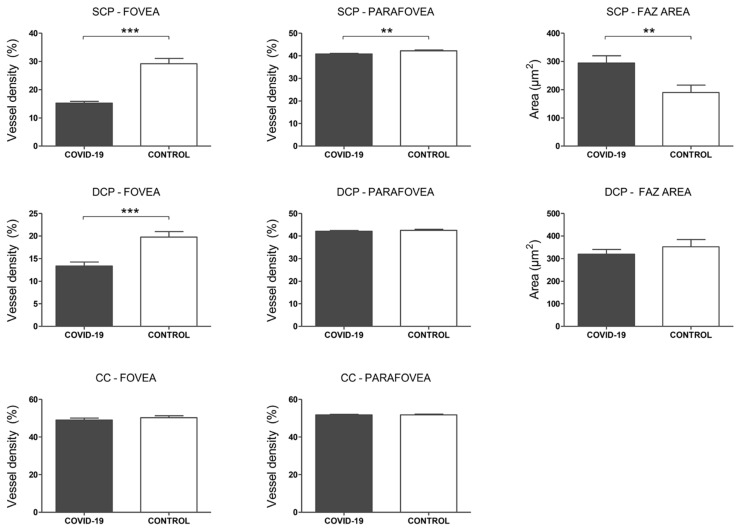
Optical coherence tomography angiography (OCTA) analysis. Top row: Superficial capillary plexus (SCP) analysis. Mean vessel density (%) of the foveal and parafoveal regions. Foveal avascular zone (FAZ) area. Middle row: Deep capillary plexus (DCP) analysis. Mean vessel density (%) of the foveal and parafoveal regions. Foveal avascular zone (FAZ) area. Bottom row: Choriocapillaris (CC) analysis. Mean vessel density (%) of the foveal and parafoveal regions. *p*-values correspond to a comparison between COVID-19 patients and controls. Error bars correspond to the SEM. ** *p* < 0.01, *** *p* < 0.001.

In the SCP, a significantly lower VD was observed in COVID-19 patients compared to controls in both foveal (15.2 ± 0.69 vs. 29.1 ± 1.88, *p* < 0.001) and parafoveal (40.8 ± 0.31 vs. 42.2 ± 0.40, *p* = 0.009) regions. In this plexus, FAZ area was also significantly greater in COVID-19 patients than in controls (294.9 ± 25.71 vs. 190.1 ± 26.47, *p* = 0.007) (Figure 5). In the DCP, a significantly lower VD was observed in the foveal area of COVID-19 patients compared to controls (13.33 ± 0.93 vs. 19.77 ± 1.53, *p* < 0.001). However, no significant differences were found in VD in the parafoveal area (42.15 ± 0.33 vs. 42.56 ± 0.47, *p* = 0.48) or FAZ area in this plexus (319.9 ± 20.91 vs. 352.4 ± 32.14, *p* = 0.39) (Figure 5). In the CC plexus, no significant differences were observed in VD in the foveal (49.1 ± 1.01 vs. 50.3 ± 1.03, *p* = 0.40) and parafoveal areas (51.8 ± 0.25 vs. 51.8 ± 0.35, *p* =0.996) between COVID-19 cases and controls (Figure 5). 

In the subgroup analysis by presence of CWS within the COVID-19 cases cohort, none of the OCTA parameters showed differences between groups (Table 3). 

## 4. Discussion

This study describes objectively microvascular alterations in the retinal plexi, which compose the internal blood-retinal barrier, of COVID-19 patients assessed by OCTA, while no significant changes were found in the choriocapillaris. We observed decreased VD and enlarged FAZ area in the perifoveal vascular network of COVID-19 severe bilateral pneumonia patients compared to a control cohort of age and sex-matched healthy controls. Moreover, we described differences in the peripapillary RNFL thickness between COVID-19 patients with and without CWS, suggesting that these may indicate different stages of the neurovascular affection of the disease. 

The findings reported in this study suggest that OCT and OCTA parameters may be used as surrogate biomarkers of the microvascular affection of the systemic disease and may reflect the microvascular changes occurring elsewhere in other organs, as the lungs or the kidneys. If confirmed in future studies, these preliminary data may highlight the role of OCT and OCTA in the assessment of COVID-19 patients to identify different disease patterns that may drive and personalize the systemic treatment of these patients.

The rapid progression of the COVID-19 pandemic has made it essential to elucidate the physiopathology behind its wide range of clinical manifestations. An increasing number of studies suggest that COVID-19 is a microvascular disease in which endothelial cells seem to play a central role in severe cases [23]. This could be explained by the strong affinity of SARS-COV-2 for the ACE-2 expressed on the surface of endothelial cells and pericytes [1]. The retina has the highest metabolic demand of any tissue on the body, which makes the eye one of the most perfused organs [24]. This is the reason why the retina maintains a constant blood flow, even in stressful situations such as changes in blood pressure and blood gas tension, often present in patients with severe COVID-19 infection. In addition, the retinal vasculature is resistant to the influence of autonomic innervation and hormonal mediators. These particularities make the retina an ideal organ to study the local microcirculation [25].

The advent of the OCTA has made it possible to study in detail microvascular alterations, detecting changes between repeated B-scans mainly produced by blood flow and displayed as a motion contrast in a non-invasive way [13]. Therefore, when VD is decreased as in our case series, two main factors may contribute to explain this process. First, a decreased blood flow velocity in the vascular bed. As previously discussed, the retina aims to maintain perfusion constant, so foveal flow impairment may occur at a local level as a result of an obstructive event in the vessel lumen, mainly by thrombotic phenomena. Second, capillary dropout could be a consequence of increased apoptosis and pyroptosis of the endothelial cells in the context of a proinflammatory endothelium disfunction. Both of these phenomena have been suggested to occur in patients with SARS-COV-2 infection, and endothelial injury could be the underlying mechanism that might link inflammation and thrombosis in severe cases [23]. 

Postmortem studies have demonstrated microvascular thrombosis and endothelial inflammation, as well as viral elements, apoptotic bodies and inflammatory cells attached to the endothelium of small vessels in several organs [4]. In contrast to other tissues that have collateral blood vessels, retinal plexi are composed by end arteries with no anastomotic connections [24]. The boundary that delimits the FAZ is composed by these terminal vessels and therefore constitutes an area particularly susceptible to ischemic changes [26]. In fact, FAZ area enlargement has been correlated with capillary loss in different retinal vascular diseases, like diabetic retinopathy and retinal vascular occlusions [27,28]. In our study we found FAZ enlargement in the SCP of COVID-19 patients compared with control subjects. 

The inner retina receives its blood supply from the SCP and DCP, the final branches of the central retinal artery, while the choroid is responsible for nourishing the outer retina. Therefore, as a consequence of the occlusion of the terminal foveal vessels, an atrophy of the inner retina may occur. In line with this, the current study showed that the thickness of the GCL was significantly decreased in COVID-19 patients. Immunohistochemical analysis have shown that the retina (specially GCL and macroglial Müller cells), the retinal pigment epithelium (RPE), and the choroid express significative levels of ACE2, which could also justify direct non-ischemic damage to the retina [29,30]. Unlike the retina, the CC is a highly anastomosed and dense network of capillaries under neurogenic control [25]. This protects the outer retina from ischemic events and microvascular changes that would be subtler and more difficult to evidence. Among other reasons, this could explain why no significant differences in VD were found in the CC.

**Figure 6:**
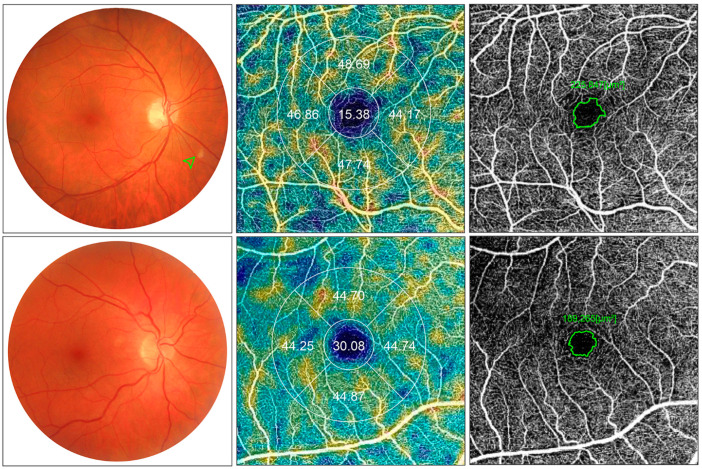
Composite images of case-control comparison. Top row: COVID-19 patient with bilateral pneumonia due to SARS-COV-2 case. Bottom row: Age-, sex- and laterality-matched control. Fundus retinography (left column), optical coherence tomography angiography (OCTA) vessel density maps (middle column), and OCTA image of the superficial capillary plexus (SCP) are depicted. In this case, a cotton wool spot is presented in the fundus retinography (green arrowhead). Reduced vessel density (15.38 vs. 30.08) and enlarged foveal avascular zone (FAZ) (225.8 vs. 189.2 µm^2^) are observed in the case compared to the matched control. 4.5 × 4.5 mm en face angiogram of the SCP are shown. Manually delineated FAZ are shown in green.

Two previous studies have described retinal alterations in fundus examination of COVID-19 patients [16,18]. Both described the presence of CWS in some of the patients; in our sample 17% of the COVID-19 patients presented these lesions in at least one eye (Figure 6). CWS are axoplasmic debris at the level of the RNFL resulting from axoplasmic flow interruption that could be due to ischemia, embolisms, connective tissue disorders, neoplasms, and infections, but sometimes no underlying cause can be identified [31,32]. The presence of this CWS might suggest that diffuse subclinical affection of the RNFL could also be happening. In fact, Burgos-Blasco et al. recently described thickening of the peripapillary RNFL in COVID-19 patients, but no CWS were found on their sample [15]. We have also found a significant thickening of the RNFL, which was greater in patients who presented visible CWS after fundoscopy exam, when comparing them with patients without these lesions, even though the CWS were not located on the quantified area. Among other retinal manifestations, an enlargement of the macrovasculature, that was directly correlated with the severity of the infection, has been described in the SERPICO-19 study [12].

Recently, other authors have studied the retinal microvasculature in patients with COVID-19 with variable results [33,34,35]. Our study reinforces previous findings of decreased VD and for the first time includes a subanalysis of subjects with CWS in patients with bilateral pneumoniae.

The COVID-19 pandemic has opened a new field of research in rapid development in which many of the published data have not yet been able to be contrasted by other authors; therefore, we must be cautious when interpreting some of these findings. In this regard, a recent study published by Marinho et al. reported hyper-reflective lesions in the GCL and inner plexiform layer (IPL) in both eyes of all the studied patients [18]. A later study from our group could not find these lesions in any of the eyes evaluated (*n* = 54) [16], and these data have not been confirmed further by any other series. Anatomically, the medium-caliber vessels from the central artery of the retina are located in the GCL and IPL [36]. The image of medium-caliber vessels has been described as hyperintense in the GCL with a prominent posterior shadow that presents higher contrast in the inner retina [37]. The hyper-reflective lesions reported in the abovementioned study could be included within this description, as they are located more frequently in the papillomacular area, where the horizontal arrangement of the vessels causes longitudinal cross-sections of the vessels, as they present the same orientation as the horizontal B-scan. Interestingly, in our sample of COVID-19 patients, hyper-reflective images very similar to those described in the study were obtained by B-scans of longitudinal cross-sections of medium-caliber retinal vessels. We consider that these lesions could correspond to normal retinal vessels, as suggested also by other authors [19,20].

The clinical relevance of the reported findings may extend beyond the ocular phenotypes of COVID-19 and may reflect the microvascular changes occurring somewhere else in the body, affecting other organs such as the brain, the lungs, or the kidneys. In keeping with this idea, from an embryological point of view, the retina is an extension of the brain, and consequently there is a significant homology on the anatomy and regulatory processes of their macro- and micro-vasculature [24]. That is why it is possible that the previously described alterations could also be occurring in the brain, which is supported by the existing evidence of cerebrovascular disease in patients with severe COVID-19 [38,39]. 

These associations between ocular and systemic status of the disease may have a direct impact in the management of these patients. A recently published study described three different profiles of disease (inflammatory, co-infection, and thrombotic profiles) in bloods patterns of COVID-19 patients using an artificial-intelligence algorithm that helps direct treatment in a personalized approach reducing significantly the mortality rate of these patients [40]. In this scenario, the information provided by OCTA about the microvascular status of the disease appears interesting, as this constitutes an accessible, fast, economic, and non-invasive test that could help in the categorization of the patients toward a personalized care.

We acknowledge a series of limitations in our study. First, the small sample size of the COVID-19 bilateral pneumonia cases cohort, as a consequence of the strict inclusion criteria applied, the use of only one study eye per patient to avoid bilaterality bias, and the reduced number of patients discharged from the hospital with negative SARS-COV-2 tests willing to attend an ophthalmic check. Second, the cross-sectional nature of the study, that prevents the ability to monitor the evolution of the retinal alterations found over time. Similarly, we do not have baseline data of the pre-infection status of the study eyes to perform a comparison with the current status of the lesions. To evaluate their evolution further in time, a longitudinal study of prospective follow-up is currently ongoing in our center. Third, there was a greater number of COVID-19 patients than controls with blood hypertension (11 vs. 5 eyes) and dyslipidemia (12 vs. 2 eyes) at the moment of the scan. At the same time, both may be independently related to the recent viral infection that caused bilateral pneumonia and required hospital admission, and these factors may have contributed at least in part to explain the differences observed. Also, our results may not be applicable to younger patients, as our study cohort mean age may reflect a population at potential risk for certain ocular diseases. Fourth, our control group included healthy volunteers, but it would have been interesting to compare our findings with postrecovery pneumonia patients. Nevertheless, we are not aware of any specific OCTA finding in such cases. Finally, fifth, we evaluated recovered patients 14 days after hospital discharge, instead during the active infection to study the acute changes on the retina. However, due to the strict recommendations of the Ophthalmological Societies for eye care [41,42], the latter option was not feasible during the first wave of the pandemic in our country, and our approach allowed us to obtain images at an early postrecovery phase, which may be an adequate option to estimate the presence of the retinal alterations in SARS-COV-2 infection. However, at present, it is known that the risk of contagion in healthcare personal is low if the recommended preventive measures are used, and we consider that in this scenario, OCTA could be a useful test to determine the microvascular status of the patient and guide their systemic treatment.

## 5. Conclusions

In conclusion, this study reports decreased VD and FAZ enlargement in COVID-19 bilateral pneumoniae patients compared to age- and sex-matched controls. In addition, COVID-19 patients presented significantly thinner GCL and thicker RNFL compared to controls, and this RNFL thickening was greater in COVID-19 cases with CWS compared to those without CWS. These findings may allow for patient classification in different profiles that may help to direct personalized therapies and ultimately improve the systemic outcomes of these patients. 

## Figures and Tables

**Table 1:** Demographic, ocular, and systemic characteristics of the study population. (* not performed as per recommended protective measures during pandemic peak).

	COVID-19	Control
Number of patients, *n*	25	25
Age (mean ± SEM)	61.35 ± 2.36	60.03 ± 2.33
Females (%)	10 (40)	10 (40)
Hypertension, *n* (%)	11 (44)	5 (20)
BMI > 30, *n* (%)	4 (16)	4 (16)
Dyslipidemia, *n* (%)	12 (48)	2 (8)
LogMAR Visual acuity (mean ± SEM)	0.05 ± 0.03	0.05 ± 0.01
Spherical equivalent (D) (mean ± SEM)	−0.2 ± 0.22	+0.2 8 ± 0.27
Axial length (mm) (mean ± SEM)	(*)	23.96 ± 0.24
Cotton wool spots, *n* (%)	5 (20%)	0

**Table 2:** Comparison of structural optical coherence tomography (OCT) and OCT angiography (OCTA) parameters in COVID-19 patients and age-, sex-, and laterality-matched controls. Mean ± SEM (standard error of the mean) structural OCT and OCTA values. Retinal nerve fiber layer (RNFL), ganglion cell layer (GCL), superficial capillary plexus (SCP), foveal avascular zone (FAZ), deep capillary plexus (DCP), and choriocapillaris (CC). *p*-values correspond to a comparison between subgroups. Bold values denote statistical significance at the *p* < 0.05 level.

		COVID-19 (Eyes = 25)	Control (Eyes = 25)	*p*-Value
Foveal, central subfield	Retinal thickness (µm)	241.1 ± 3.77	239.2 ± 9.38	0.85
	RNFL thickness (µm)	7.3 ± 0.50	8.38 ± 0.86	0.28
	GCL thickness (µm)	44.8 ± 1.32	54.4 ± 2.90	0.033
	Choroid thickness (µm)	237.6 ± 17.83	250.5 ± 17.73	**0.60**
Optic nerve head	Optic RNFL thickness (µm)	111.4 ± 2.31	103.1 ± 3.45	**0.048**
Superficial capillary plexus	Foveal vessel density (%)	15.2 ± 0.69	29.1 ± 1.88	**<0.001**
	Parafoveal vessel density (%)	40.8 ± 0.31	42.2 ± 0.40	**0.009**
	FAZ area (µm^2^)	294.9 ± 25.71	190.1 ± 26.47	**0.007**
Deep capillary plexus	Foveal vessel density (%)	13.33 ± 0.93	19.77 ± 1.53	**0.001**
	Parafoveal vessel density (%)	42.15 ± 0.33	42.56 ± 0.47	0.48
	FAZ area (µm^2^)	319.9 ± 20.91	352.4 ± 32.14	0.39
Choriocapillaris	Foveal vessel density (%)	49.1 ± 1.01	50.3 ± 1.03	0.40
	Parafoveal vessel density (%)	51.8 ± 0.25	51.8 ± 0.35	0.996

**Table 3:** Subgroup analysis in COVID-19 patients with and without cotton wool spots (CWS). Mean ± SEM (standard error of the mean) structural OCT and OCTA values. Retinal nerve fiber layer (RNFL), ganglion cell layer (GCL), superficial capillary plexus (SCP), foveal avascular zone (FAZ), deep capillary plexus (DCP), and choriocapillaris (CC). *p*-values correspond to a comparison between subgroups. Bold values denote statistical significance at the *p* < 0.05 level.

		CWS (Eyes = 25)	Without CWS (Eyes = 25)	*p*-Value
Foveal, central subfield	Retinal thickness (µm)	247.2 ± 6.01	239.5 ± 3.54	0.47
	RNFL thickness (µm)	7.5 ± 0.92	7.58 ± 0.55	0.96
	GCL thickness (µm)	43.8 ± 1.20	44.5 ± 1.13	0.83
	Choroid thickness (µm)	237.5 ± 14.97	241.6 ± 26.31	0.92
Optic nerve head	Optic RNFL thickness (µm)	121.8 ± 7.62	109.0 ± 1.81	**0.032**
Superficial capillary plexus	Foveal vessel density (%)	13.95 ± 0.58	14.95 ± 0.57	0.56
	Parafoveal vessel density (%)	41.3 ± 0.85	40.67 ± 0.26	0.41
	FAZ area (µm^2^)	339.9 ± 30.28	304.0 ± 19.12	0.53
Deep capillary plexus	Foveal vessel density (%)	13.49 ± 1.61	13.29 ± 0.77	0.93
	Parafoveal vessel density (%)	43.3 ± 0.71	43.59 ± 1.46	0.77
	FAZ area (µm^2^)	331.7 ± 30.50	337.6 ± 21.68	0.92
Choriocapillaris	Foveal vessel density (%)	45.88 ± 2.29	48.5 ± 0.89	0.34
	Parafoveal vessel density (%)	50.96 ± 0.36	54.15 ± 2.40	0.66

## Data Availability

All data are available within the manuscript and upon request to corresponding author.
